# Simulated mussel mortality thresholds as a function of mussel biomass and nutrient loading

**DOI:** 10.7717/peerj.2838

**Published:** 2017-01-04

**Authors:** Jeremy S. Bril, Kathryn Langenfeld, Craig L. Just, Scott N. Spak, Teresa J. Newton

**Affiliations:** 1 Department of Civil and Environmental Engineering, University of Iowa, Iowa City, IA, United States; 2 Upper Midwest Environmental Sciences Center, US Geological Survey, La Crosse, WI, United States

**Keywords:** Native freshwater mussels, Ammonia mortality thresholds, Nutrients, Numerical modeling

## Abstract

A freshwater “mussel mortality threshold” was explored as a function of porewater ammonium (NH_4_^+^) concentration, mussel biomass, and total nitrogen (N) utilizing a numerical model calibrated with data from mesocosms with and without mussels. A mortality threshold of 2 mg-N L^−1^ porewater NH_4_^+^ was selected based on a study that estimated 100% mortality of juvenile *Lampsilis* mussels exposed to 1.9 mg-N L^−1^ NH_4_^+^ in equilibrium with 0.18 mg-N L^−1^ NH_3_. At the highest simulated mussel biomass (560 g m^−2^) and the lowest simulated influent water “food” concentration (0.1 mg-N L^−1^), the porewater NH_4_^+^ concentration after a 2,160 h timespan without mussels was 0.5 mg-N L^−1^ compared to 2.25 mg-N L^−1^ with mussels. Continuing these simulations while varying mussel biomass and N content yielded a mortality threshold contour that was essentially linear which contradicted the non-linear and non-monotonic relationship suggested by Strayer (2014). Our model suggests that mussels spatially focus nutrients from the overlying water to the sediments as evidenced by elevated porewater NH_4_^+^ in mesocosms with mussels. However, our previous work and the model utilized here show elevated concentrations of nitrite and nitrate in overlying waters as an indirect consequence of mussel activity. Even when the simulated overlying water food availability was quite low, the mortality threshold was reached at a mussel biomass of about 480 g m^−2^. At a food concentration of 10 mg-N L^−1^, the mortality threshold was reached at a biomass of about 250 g m^−2^. Our model suggests the mortality threshold for juvenile *Lampsilis* species could be exceeded at low mussel biomass if exposed for even a short time to the highly elevated total N loadings endemic to the agricultural Midwest.

## Introduction

Native freshwater mussels are large (25–200+ mm in length), long-lived (>25 y) invertebrates that transfer nutrients from the overlying water to sediments through filter feeding (Christian et al., 2005). These benthic, burrowing, and suspension-feeding bivalves stimulate production across multiple trophic levels (Vaughn, Nichols & Spooner, 2008); the biomass of healthy mussel beds can exceed the biomass of all benthic organisms by an order of magnitude (Negus, 1966; Layzer Gordon & Anderson, 1993). There are billions of mussels within the Upper Mississippi River (UMR) and the filtration capacity in a 480 km segment (about 13% of the river length), as a percentage of river discharge, is estimated to be up to 1.4% at high flows, up to 4.4% at moderate flows, and up to 12.2% during low flows (Newton et al., 2011). Collectively, these mussels filter over 14 billion gallons of water, remove tons of particulate organic matter from the overlying water, and deposit tons of ammonium (NH${}_{4}^{+}$), associated ammonia (NH_3_), and carbon at the sediment-water interface each day.

**Figure 1:**
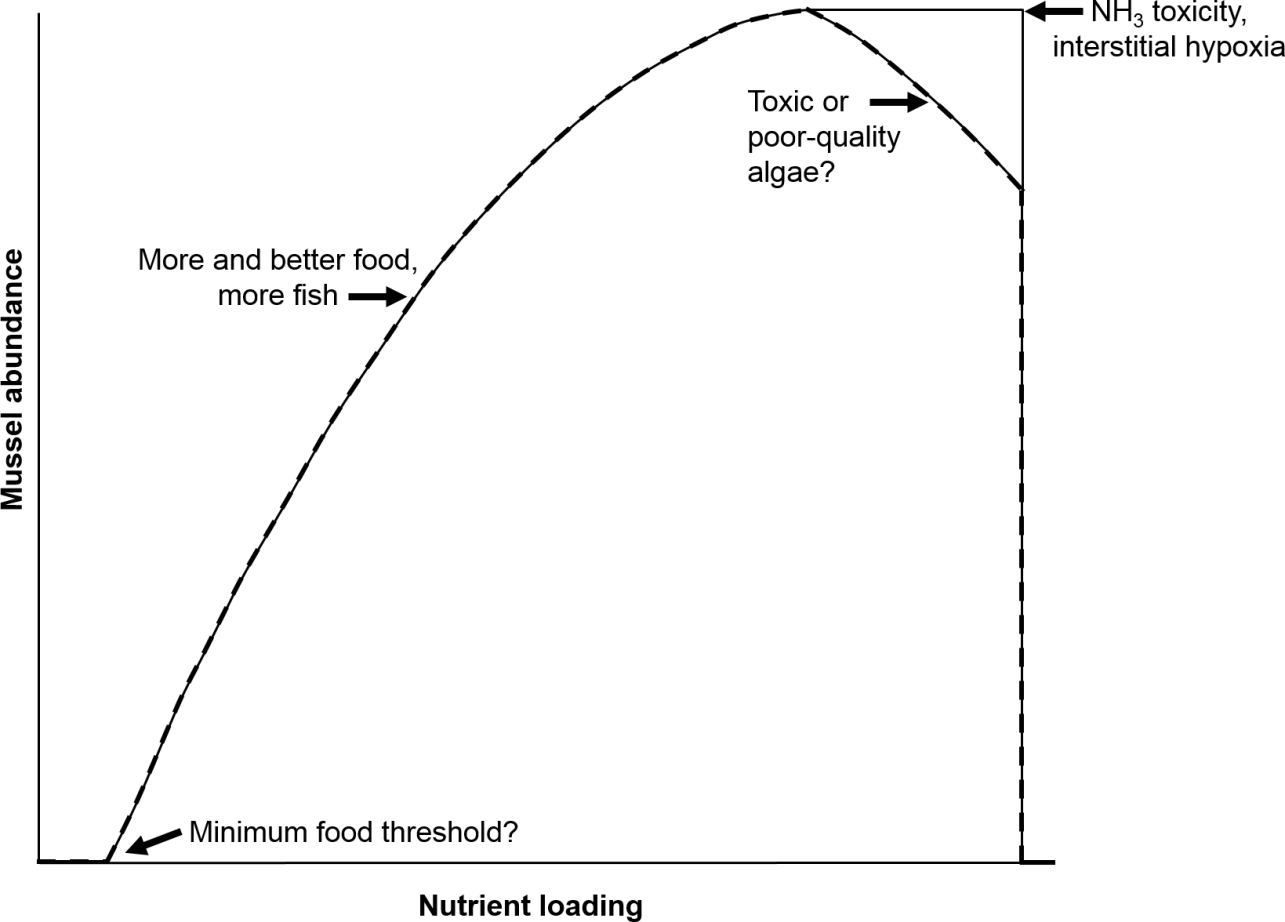
Hypothetical relationship between nutrient loading and mussel abundance. Concepts of minimum food threshold, ammonia toxicity, etc. are postulated to define the displayed curve. Adapted from Strayer (2014).

Our previous work showed that native freshwater mussels directly elevate NH${}_{4}^{+}$ and indirectly elevate nitrate (NO${}_{3}^{-}$) and nitrite (NO${}_{2}^{-}$) concentrations in lab-based mesocosms (Bril et al., 2014). The increase in NH${}_{4}^{+}$ concentrations by mussels has been associated with ingestion of food (e.g., algae, phytoplankton, bacteria, and fungi), digestion, and subsequent NH${}_{4}^{+}$ excretion (Thorp et al., 1998; Vaughn, Nichols & Spooner, 2008). However, the dynamics among food, mussels, NH${}_{4}^{+}$, and, more broadly the nitrogen (N) cycle, especially given increasing anthropogenic releases of nutrients to mussel habitats, remain poorly understood (Strayer, 2014). The negative aspects of increased nutrient loading are most frequently reported, but an increase in nutrients to some level, may favor growth and fecundity and may increase populations of host fish (Strayer, 2014). However, there is likely a threshold, such that extreme eutrophication may have negative consequences for mussels, perhaps by decreasing the fatty acid content of food (Muller-Navarra et al., 2004; Basen Martin-Creuzburg & Rothhaupt , 2011) and/or by increasing levels of toxic *Microcystis* algae (Bontes et al., 2007). These realities led us to examine where the biogeochemical boundaries and thresholds are that indicate healthy versus unhealthy outcomes for freshwater mussels as a function of variable nutrient loadings and mussel biomass. A hypothetical relationship between mussel abundance and nutrient loading has been proposed by Strayer (2014) (Fig. 1), that postulates thresholds for minimum food, NH_3_ toxicity, interstitial hypoxia and toxic or poor algae quality. Strayer concluded that “it would be useful to identify early warning signs that the ‘death threshold’ is about to be crossed.” Thus, the objective of our study was to develop a numerical model to conceptualize this “mortality threshold” as governed by mussel biomass and nutrient loading.

Little is known about minimum food thresholds (let alone food quality guidelines) for mussels and, in the current era of increasing nutrient loadings, this concept will likely become less relevant over time (Bergstrom & Jansson, 2006; Strayer, 2014). Therefore, we chose elevated porewater NH${}_{4}^{+}$ concentration as an easily measured indicator of potential mortality thresholds for mussels. This is biologically relevant because native freshwater mussels have been shown to be some of the most sensitive organisms tested for NH_3_ toxicity associated with equilibrium concentrations of NH${}_{4}^{+}$ (Augspurger et al., 2003; Newton & Bartsch, 2007). A fraction of the toxic biological response, regardless of species, is almost certainly caused by NH_3_ in equilibrium with NH${}_{4}^{+}$. Therefore, NH${}_{4}^{+}$ concentration is an acceptable surrogate for total ammonia nitrogen only when the temperature and pH of the aquatic habitat is known. The deposition of NH${}_{4}^{+}$ and other reduced N species by mussels comes mostly in the form of feces and pseudofeces (Vaughn, Gido & Spooner, 2004; Lauringson et al., 2007; Christian, Crump & Berg, 2008; Gergs, Rinke & Rothhaupt, 2009). About 90% of the food taken in by mussels is excreted (Christian, Crump & Berg, 2008), which emphasizes the importance of knowing food concentrations, especially as a function of N content, when predicting associated porewater NH${}_{4}^{+}$ concentrations.

This study focuses on an intensively sampled 10-d data set that was used to evaluate the ability of our numerical model to simulate food, NH${}_{4}^{+}$, NO${}_{2}^{-}$, NO${}_{3}^{-}$, organic N (org N), and total N concentrations in the overlying water and porewater of continuous-flow laboratory mesocosms. The model was calibrated using literature values and water chemistry measurements from a separate, 7-d mesocosm sampling period reported in our previous work (Bril et al., 2014). The mussel species *Amblema plicata* and *Lampsilis cardium* were selected due to their abundance in the Iowa River (Zohrer, 2006) and throughout the UMR Basin (Newton et al., 2011), where N runoff from industrial agriculture severely impacts the aquatic N cycle. This research is novel in that a multi-rate nitrification/denitrification model was developed, calibrated, and evaluated with sensor-based, highly time-resolved data from mesocosms containing mussels. To our knowledge, this is the first use of such a model to simulate various “mortality threshold” scenarios for mussels.

## Materials and Methods

### Mesocosm setup

**Figure 2:**
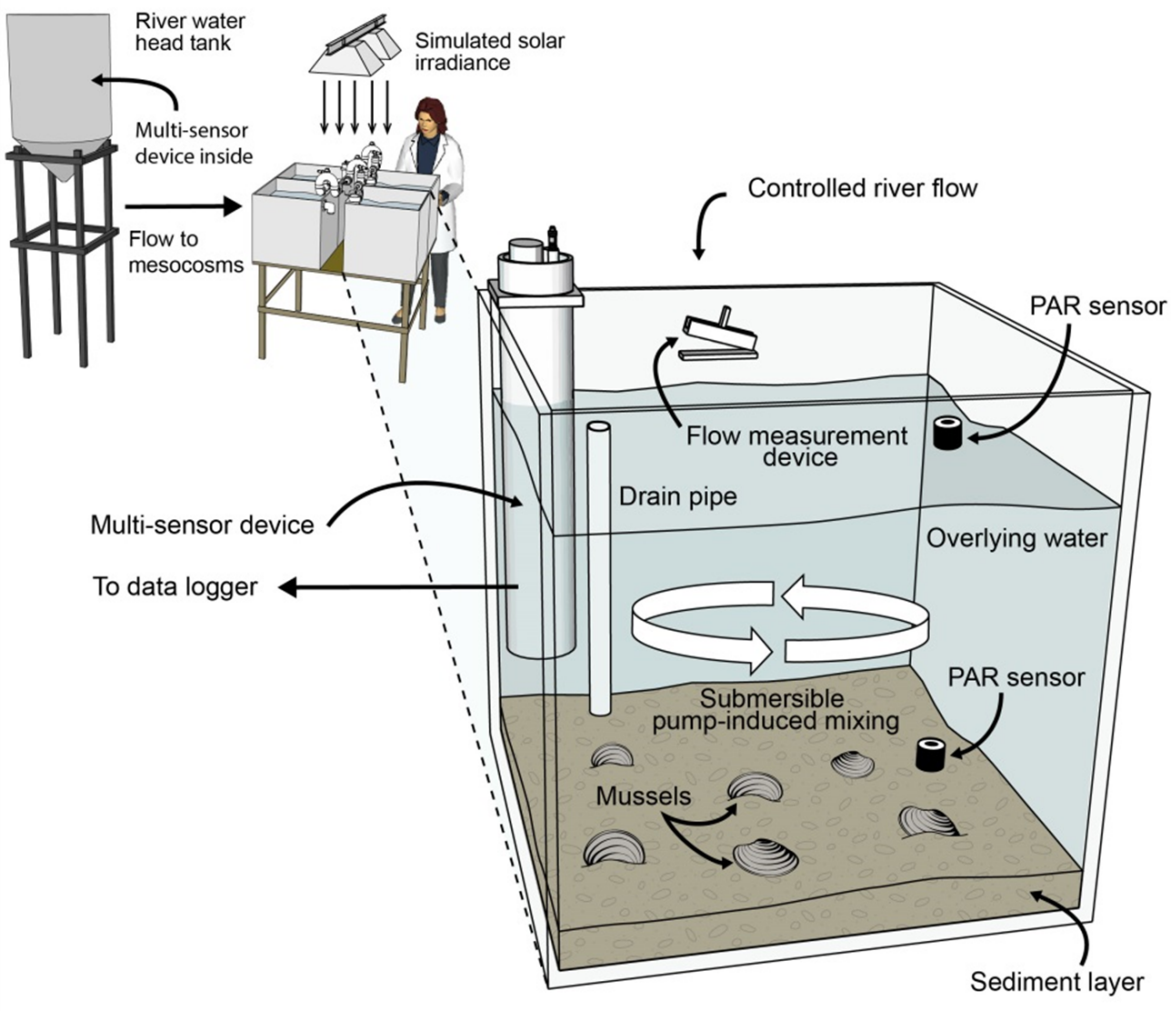
Schematic diagram of the flow-through, 4-mesocosm system, which was continuously fed Iowa River water (monitored with a multisensor device), contained a sand and river-sediment bottom layer and was irradiated with simulated sunlight (12 h daily). Each mesocosm was equipped with a constant head inlet, a flow measurement device, a recirculating pump, photosynthetically active radiation (PAR) sensors, and a multisensor, water-chemistry device. Two mesocosms contained mussels, and 2 contained no mussels.

Four 140 L, flow-through mesocosms (Fig. 2) continuously received untreated Iowa River water during the 107-d experiment, which culminated in an intensive 10-d water chemistry sampling period. Two mesocosms contained mussels collected from the Iowa River and two were without mussels (control). Twelve adult *A. plicata* and 13 adult *L. cardium* were placed in one mesocosm and 13 *A. plicata* and 12 *L. cardium* were placed in another mesocosm. This approximates a density of 70 mussels m^−2^, which although high, is still a realistic density in some reaches of the UMR (Newton et al., 2011). Across both mesocosms, shell length (±1 standard deviation) was 95 ± 20 mm in *A. plicata* and 120 ± 25 mm in* L. cardium*. Initially, all mesocosms contained 8 cm of purchased sand substrate, but particulate deposition from the river water altered this composition over time. A gravity-fed, constant head system provided a controllable flow rate between 9 and 55 L h^−1^. The flow rate during the 10-d intensive sampling period was 8.5 L h^−1^ (16 h hydraulic residence time). Complete mixing in each mesocosm was provided by 1,500 L h^−1^ submersible pumps, and two 1,000-watt solar simulators provided illumination on a 12:12 h light–dark cycle. Additional details regarding the mussel mesocosm system are available elsewhere (Bril et al., 2014).

### Mesocosm sampling and analyses

**Figure 3:**
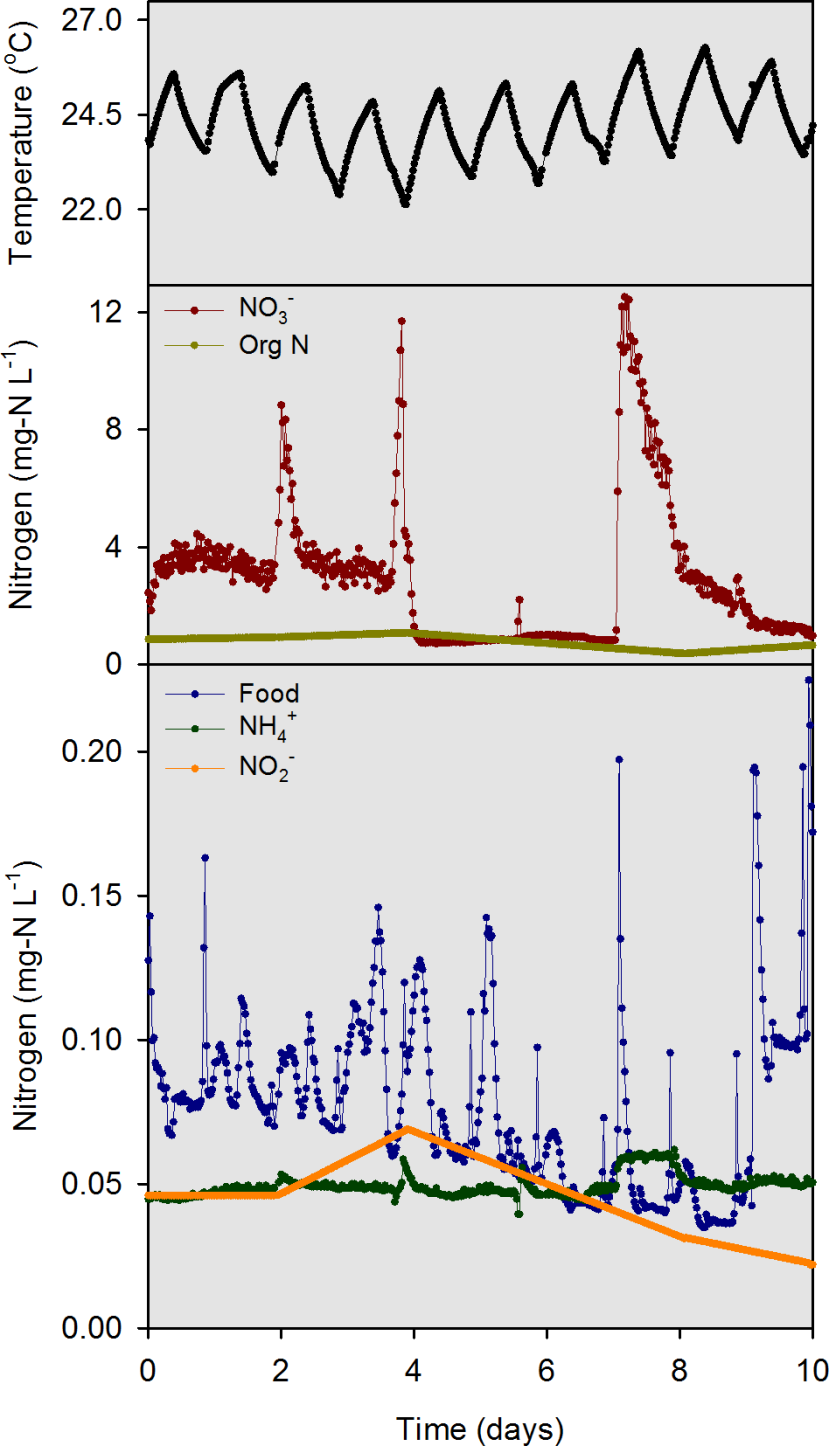
Model input data for temperature, food (converted from chl *a* data), NH${}_{4}^{+}$, NO${}_{2}^{-}$, NO${}_{3}^{-}$, and org N as measured in the river water head tank during the 10-d model evaluation period.

Data from a 10-d intensive sampling period (days 97–107 of the 107-d experiment) were used for model evaluation. We intentionally delayed the start of the intensive sampling by 97 days so that the mussels could acclimate and bacteria responsible for nitrification and denitrification could establish. Electronic water chemistry sensors (model DS5; Hach Chemical Company, Loveland, CO, USA) were used to measure highly time-resolved (30-min) water chemistry data in the overlying water of each mesocosm and in the influent head tank. The sensors measured chlorophyll *a* (chl-*a*), NH${}_{4}^{+}$, NO${}_{3}^{-}$, pH, and temperature. Custom-made flow measurement devices with magnetic reed switches were used to quantify influent flow. Photosynthetically active radiation (PAR) sensors (model SQ-120; Apogee Instruments, Logan, Utah) were used to measure solar irradiance at the substrate and water surface of each mesocosm. All measurements obtained by the sensors were collected and stored using two data loggers. The model inputs for influent river temperature, food, NH${}_{4}^{+}$, NO${}_{2}^{-}$, NO${}_{3}^{-}$ and org N (Fig. 3) were measured values from within the river water head tank during the 10-d sampling period.

Discrete water chemistry samples were collected and analyzed at five time points during the 10-d sampling period from the overlying water and porewater of each mesocosm and from the influent head tank. The discrete samples were analyzed for chl-*a*, NH${}_{4}^{+}$, NO${}_{2}^{-}$, NO${}_{3}^{-}$, org N, and total N. Chl-*a* was measured by fluorescence. Measured chl-*a* concentrations (µg L^−1^) were converted to “food” biomass (mg L^−1^) based on literature values for phytoplankton chl-*a* content (Kasprzak et al., 2008). The fraction of nitrogen in food biomass (mg-N L^−1^) was calculated using the empirical formula C_106_H_263_O_110_N_16_P (Chapra, 1997). NH${}_{4}^{+}$ was determined using the Salicylate Method, and NO${}_{3}^{-}$ was determined using the Dimethylphenol Method (APHA, 1996). NO${}_{2}^{-}$ was measured using the Diazotization Method, and total N was measured using the Persulfate Digestion Method (APHA, 1996). Sample measurements for org N were estimated by subtracting the sum of NH${}_{4}^{+}$, NO${}_{3}^{-}$, and NO${}_{2}^{-}$ from the total N measurements. A more detailed description of the mesocosm sampling and analysis setup is available (Bril et al., 2014).

### Model calibration and sensitivity analyses

Seven days of the 107-d experiment were intensively sampled and previously reported (Bril et al., 2014) for food, NH${}_{4}^{+}$, NO${}_{2}^{-}$, NO${}_{3}^{-}$, org N, and temperature; these values were used as model calibration inputs. Linear interpolation between discrete samples was used where 30-min measurements were unavailable (org N, NO${}_{2}^{-}$, and total N), and ranges for unmeasured model variables (e.g., nitrification rate, denitrification rate) were obtained from the literature (Table 1). The model, created in Stella (version 8.0, ISEE Systems, Inc., Lebanon, New Hampshire), was initially calibrated using the no-mussel control data, then refined using data from mesocosms containing mussels to properly parameterize clearance and excretion rates (Bayne, Hawkins & Navarro, 1987; Englund & Heino, 1994; Haag, 2012). The optimized values used in the model calibration are given in Table 1. The optimized calibration values were determined by comparing model outputs to sensor and discrete sample measurements and then minimizing normalized mean error and maximizing *R*^2^ values (Table 2).

**Table 1:** Model calibration values.

Variable	Description	Literature range	Calibration
H	Water depth (m)	–	0.406
k_ai_(T)	NH${}_{4}^{+}$ to NO${}_{2}^{-}$ nitrification rate (h^−1^)	–	0.12
k_am_	Half-saturation constant for NH${}_{4}^{+}$ preference (mg-N L^−1^)	0.001–0.05 (Chapra, 1997)	0.05
k_d_(T)	Food death rate (h^−1^)	0.0021–0.0104 (Schnoor, 1996)	0.002
k_dn_(T)	Denitrification rate (h^−1^)	0.0005–0.0996 (Richardson et al., 2004)	0.0005
k_g,T_	Food growth rate (h^−1^)	0.0417–0.0833 (Schnoor, 1996)	0.025
k_hn_(T)	Org N hydrolysis rate (h^−1^)	0.00004–0.0083 (Schnoor, 1996)	0.00004
k_ig_(T)	NO${}_{2}^{-}$ to N_2_ gas denitrification rate (h^−1^)	–	0.0005
k_in_(T)	NO${}_{2}^{-}$ to NO${}_{3}^{-}$ nitrification rate (h^−1^)	–	0.21
k_n_(T)	Nitrification rate (h^−1^)	0.0001–0.21 (Strauss et al., 2004)	0.1
k_ni_(T)	NO${}_{3}^{-}$ to NO${}_{2}^{-}$ denitrification rate (h^−1^)	–	0.0005
k_ra_(T)	Food respiration/excretion rate (h^−1^)	0.0004–0.0208 (Chapra, 1997)	0.004
k_sn_	Nitrogen half-saturation constant (mg-N L^−1^)	0.005–0.02 (Chapra, 1997)	0.02
k_sp_	Phosphorus half-saturation constant (mg-P L^−1^)	0.001–0.005 (Chapra, 1997)	0.005
M_b_	Mussel biomass (g)	–	200
M_cl_	Mussel clearance rate (h^−1^ g^−1^ mussel biomass)	0.000007–0.00786 (Silverman et al., 1997; Spooner & Vaughn, 2008; Newton et al., 2011)	0.002^a^, 0.0005^b^
M_ex_	Mussel excretion rate (mg-N L^−1^ h^−1^ g^−1^ mussel biomass)	0.0001–0.00083 (Baker & Hornbach, 2000; Baker & Hornbach, 2001; Christian et al., 2008; Spooner & Vaughn, 2008)	0.00009^a^, 0.000075^b^
p	Phosphorus concentration (mg-P L^−1^)	0.04–1.31 (Espinosa-Villegas et al., 2004)	0.3
T	Temperature (°C)	5–25 (Espinosa-Villegas et al., 2004)	Variable
V_s,a_	Food + settling rate (m h^−1^)	0–0.083 (Schnoor, 1996)	0.001
V_s,o_	Org N settling rate (m h^−1^)	0–0.083 (Schnoor, 1996)	0.001
*τ*	Hydraulic retention time (h)	–	2.5
*φ*_L_	Light attenuation factor	0–1 (Steele, 1965)	Variable

**Notes.**

a Value used when food concentration > 0.1 mg-N L^−1^, and hydraulic retention time < 12 h.

b Value used when food concentration ≤ 0.1 mg-N L^−1^, and hydraulic retention time < 12 h.

**Table 2:** Model performance statistics.

Measurement type	Parameter	Concentration (mg-N L^−1^)				Mean bias (mg-N L^−1^)	Mean error (mg-N L^−1^)	NMB	NME	*R*^2^	RMSE
		Observed		Simulated							
		Mean	SD	Mean	SD						
7-d model calibration											
Sensor	Food	0.07	0.03	0.07	0.04	0.004	0.013	5.2%	20%	0.81	0.018
	NH${}_{4}^{+}$	0.09	0.01	0.09	0.01	−0.001	0.005	−1.3%	6%	0.33	0.006
	NO${}_{3}^{-}$	0.62	0.11	0.62	0.10	0.0001	0.024	0.02%	4%	0.94	0.030
Discrete sample	Food	0.07	0.03	0.07	0.03	−0.007	0.037	−9.9%	51%	0.01	0.045
	NH${}_{4}^{+}$	0.09	0.02	0.09	0.01	−0.002	0.012	−2.4%	13%	0.10	0.015
	NO${}_{3}^{-}$	0.61	0.14	0.61	0.11	0.004	0.034	0.6%	6%	0.91	0.048
	Org N	0.49	0.13	0.49	0.15	0.002	0.118	0.3%	24%	0.19	0.142
	NO${}_{2}^{-}$	0.05	0.01	0.04	0.01	−0.002	0.006	−5.2%	12%	0.37	0.006
	Total N	1.2	0.18	1.2	0.19	−0.00004	0.111	−0.003%	9%	0.54	0.133
10-d model evaluation											
Sensor	Food^a^	0.08	0.03	0.08	0.03	−0.013	0.013	−17.1%	17.1%	0.85	0.016
	NH${}_{4}^{+}$	0.03	0.002	0.03	0.002	0.0003	0.001	1.0%	7.7%	0.35	0.001
	NO${}_{3}^{-}$	3.5	1.8	3.0	1.3	−0.513	0.549	−14.5%	15.5%	0.93	0.817
Discrete sample	Food	0.03	0.02	0.09	0.04	0.064	0.065	250%	260%	0.51	0.080
	NH${}_{4}^{+}$	0.04	0.01	0.03	0.002	−0.004	0.011	−11%	28%	0.06	0.014
	NO${}_{3}^{-}$	4.3	2.5	3.5	1.5	−0.874	0.938	−20%	22%	0.98	1.391
	Org N	0.79	0.14	0.77	0.24	−0.027	0.090	−3.4%	11%	0.78	0.121
	NO${}_{2}^{-}$	0.03	0.01	0.03	0.005	0.001	0.006	3.1%	19%	0.62	0.006
	Total N	5.2	2.5	4.3	1.5	−0.903	0.954	−17%	18%	0.96	1.296

**Notes.**

a 25 day moving average.

SD Standard deviation NMB Normalized mean bias NME Normalized mean error RMSE Root mean square error

Sensitivity analyses were conducted to identify the most important variables contributing to net system dynamic concentration response. A single variable sensitivity analysis was completed by adjusting the model variables based on a range of literature values (Table 1). When such information was unavailable, the value of the variable used in model calibration was adjusted by ±50%. Ten sensitivity model runs were completed for each variable using values obtained by sampling the range of literature values (or ±50% adjustments) at 10 equal intervals. The sensitivity analysis was considered for the normalized sensitivity coefficient (Fasham, Ducklow & McKelvie , 1990) (NSC):

(1) \begin{eqnarray*}\mathrm{NSC}= \left( \frac{ \frac{\varphi }{{\varphi }_{0}} }{ \frac{P}{{P}_{0}} } \right) \frac{{P}_{0}}{{\varphi }_{0}} \end{eqnarray*}

where, *φ* = mean value of a parameter (e.g., NH${}_{4}^{+}$, NO${}_{3}^{-}$) over the simulation period for the sensitivity run (mg-N L^−1^), *φ*_*o*_ = mean value of a parameter over the simulation period for the calibrated model (mg-N L^−1^), *P* = value of model variable in sensitivity run, and *P*_*o*_ = value of model variable in calibrated model. The NSC values for each sensitivity run were averaged to determine a net NSC for each model variable.

### Mussel mortality threshold simulations

Based on 28-day laboratory toxicity tests with juvenile fat mucket mussels (*Lampsilis siliquoidea*), Wang et al. (2011) reported that 100% mortality occurred at 2.08 mg L^−1^ total ammonia nitrogen (TAN). Given the pH (8.2) and temperature (20 °C) of that study, of the 2.08 mg L^−1^TAN, about 1.9 mg-N L^−1^ would be in the NH${}_{4}^{+}$ form and about 0.18 mg-N L^−1^ would be in the NH_3_ form. Given that our models were developed at a similar pH (8.2) and temperature (24 °C) to the Wang et al. (2011) study, we selected 2.0 mg-N L^−1^ NH${}_{4}^{+}$ in porewater as a surrogate mortality threshold for *Lampsilis* mussels. Furthermore, the US Environmental Protection Agency (EPA) determined species mean chronic values of NH_3_ for *Lampsilis siliquoidea* and *L. fasciola* to calculate a geometric mean chronic NH_3_ value of 2.1 mg-N L^−1^ for the genus *Lampsilis* (US Environmental Protection Agency, 2013).

The average measured porewater concentrations for NH${}_{4}^{+}$, NO${}_{3}^{-}$, NO${}_{2}^{-}$, org N, and food during the 10-d evaluation period (3.9, 0.2, 0.06, 5, and 0.1 mg-N L^−1^, respectively) were used as initial conditions for porewater in the model. The average overlying water concentrations for the same variables were 0.05, 5, 0.05, 2.8, and 0.1 mg-N L^−1^, respectively, and the “river water” inputs for 90-d model simulations were initially set to these values. The mussel density in our mesocosms was converted to estimated biomass (g m^−2^) using the allometric function, M = aL^b^, where M is tissue dry mass (g) and L is length (mm) and with values for “a” and “b” for *A. plicata* taken from the literature (Newton et al., 2011). The resulting mass of 6.0 g mussel^−1^ was multiplied by 35 mussels m^−2^ (half the population) to determine an estimated biomass of 210 g m^−2^ for *A. plicata.* In the absence of allometric data for *L. cardium*, the tissue dry mass was assumed to be 10 g mussel^−1^(167% of *A. plicata*), and when multiplied by 35 mussels m^−2^ resulted in a biomass of 350 g m^−2^. Adding these values gave a maximum biomass of 560 g m^−2^,  which was used as the upper bound for the simulations. To simulate changes in porewater NH${}_{4}^{+}$ concentration as a function of mussel biomass and food availability, mussel biomass was varied at zero, 140, 280, 420 and 560 g m^−2^ while the N content of food was varied at zero, 0.1, 1, 5 and 10 mg-N L^−1^.

## Results and Discussion

### Model Evaluation

**Figure 4:**
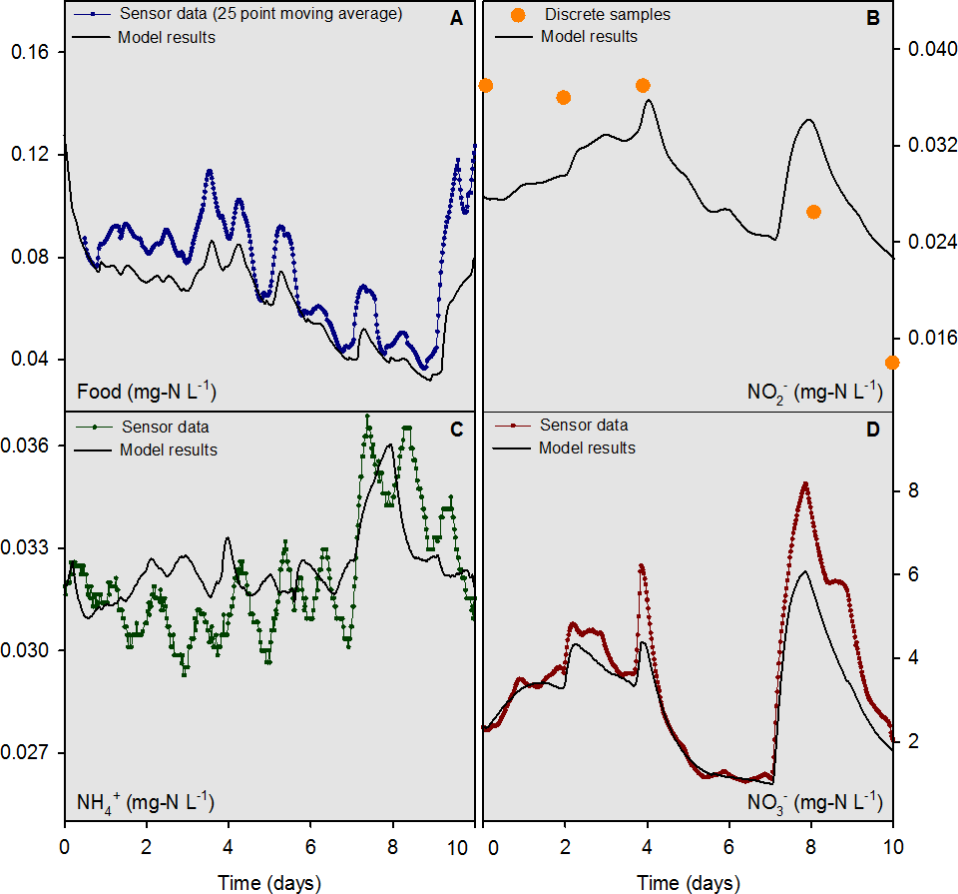
Overlying water sensor data and discrete sample results from the mesocosms containing mussels compared to model outputs for food, NH${}_{4}^{+}$, NO${}_{2}^{-}$, and NO${}_{3}^{-}$ for the 10-d model evaluation period.

For the river water head tank (pH 8.2), a combination of sensor data (temperature, NO${}_{3}^{-}$, “food,” and NH${}_{4}^{+}$) and interpolated discrete data (org N and NO${}_{2}^{-}$) were collected and used as input to the numerical model on a 30 min time step (Fig. 3). For overlying water in mesocosms, the “food” sensor data were converted to a 25-d moving average (Fig. 4A) to condition the inherently noisy signal to enable visual comparison to the model output. The discrete sample results for NO${}_{2}^{-}$ concentrations in the overlying water were similar in magnitude, but did not agree closely with the model output (Fig. 4B). The model output for NH${}_{4}^{+}$ and NO${}_{3}^{-}$ concentrations (Figs. 4C and 4D) compared well with the sensor measurements. Overall, the model was capable of outputting results that accurately predicted the concentrations, and most of the dynamics, of the major N species at a 30 min time interval for the 10-d evaluation period.

The model was evaluated quantitatively using the standard deviation (SD) of the measured data variable compared to the root mean square error (RMSE) of the model output. If the RMSE was less than half the SD, the model output for that variable was deemed “accurate” (Singh et al., 2005; Moriasi et al., 2007). For comparative purposes, values for the mean bias, mean error, normalized mean bias, normalized mean error, and *R*^2^ are reported along with the SD and RMSE for food, NH${}_{4}^{+}$, NO${}_{2}^{-}$, NO${}_{3}^{-},$ org N, and total N for the 7-d model calibration and 10-d evaluation periods (Table 2). The RMSE to SD ratio was ≤0.5 for the sensor-measured data for food, NH${}_{4}^{+}$ and NO${}_{3}^{-}$ for the 10-d evaluation period. The model evaluation based on discrete sample data yielded mixed results with RMSE to SD ratios of 0.55, 0.60, and 0.52 for NO${}_{3}^{-}$, NO${}_{2}^{-}$ and total N, respectively. The RMSE to SD ratios for food, NH${}_{4}^{+}$, and org N were 4.0, 1.4, and 0.86 for the discrete sample data, respectively. The lower accuracy determinations based on discrete sample data were likely a function of the small sample sizes, as compared to sensor measurements, and the low concentrations of food and NH${}_{4}^{+}$ which challenged the analytical limits of quantitation for these variables.

### Sensitivity analysis

The modeled nitrogen species were collectively most sensitive to changes in temperature, hydraulic retention time, and mussel biomass (Table 3). Temperature was expected to be an influential variable since the majority of the first-order rate expressions are temperature dependent. Hydraulic retention time was also expected to be influential since the influent river water has a major impact on mesocosm water chemistry in a continuous-flow system. Mussel biomass was an unexpectedly sensitive model variable. However, given the influence of mussels on food, NH${}_{4}^{+}$, NO${}_{2}^{-}$, and NO${}_{3}^{-}$ concentrations shown in our previous work (Bril et al., 2014), this result, in hindsight, should have been anticipated.

**Table 3:** Most influential variables for simulated parameters (in decreasing order).

Food	NH${}_{4}^{+}$	NO${}_{2}^{-}$	NO${}_{3}^{-}$	Org N	Total N
Temperature	Mussel excretion rate	NH${}_{4}^{+}$ to NO${}_{2}^{-}$ rate	Temperature	Water depth	Temperature
Mussel biomass	Mussel biomass	NO${}_{2}^{-}$ to NO${}_{3}^{-}$ rate	Hydraulic retention time	Org N settling rate	Mussel biomass
Hydraulic retention time	Nitrification rate	Temperature	Nitrification rate	Hydraulic retention time	Mussel excretion rate

### Mussel mortality threshold simulations

**Figure 5:**
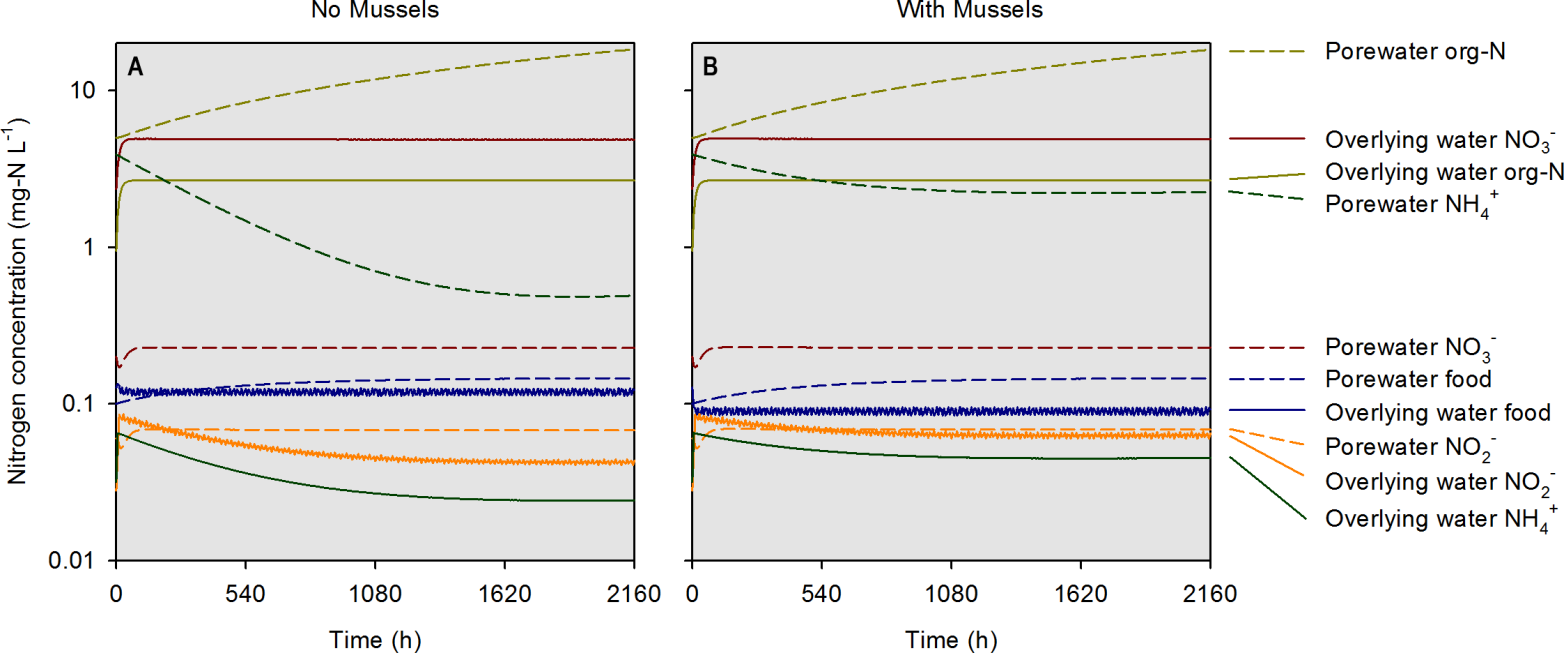
Simulated concentrations of various nitrogen-containing species over a 2,160 h (90 d) timespan in the absence and presence of mussels at a specific biomass (560 g m^−2^). Modeled constituents in porewater and overlying water are shown by dashed and solid lines, respectively.

**Figure 6:**
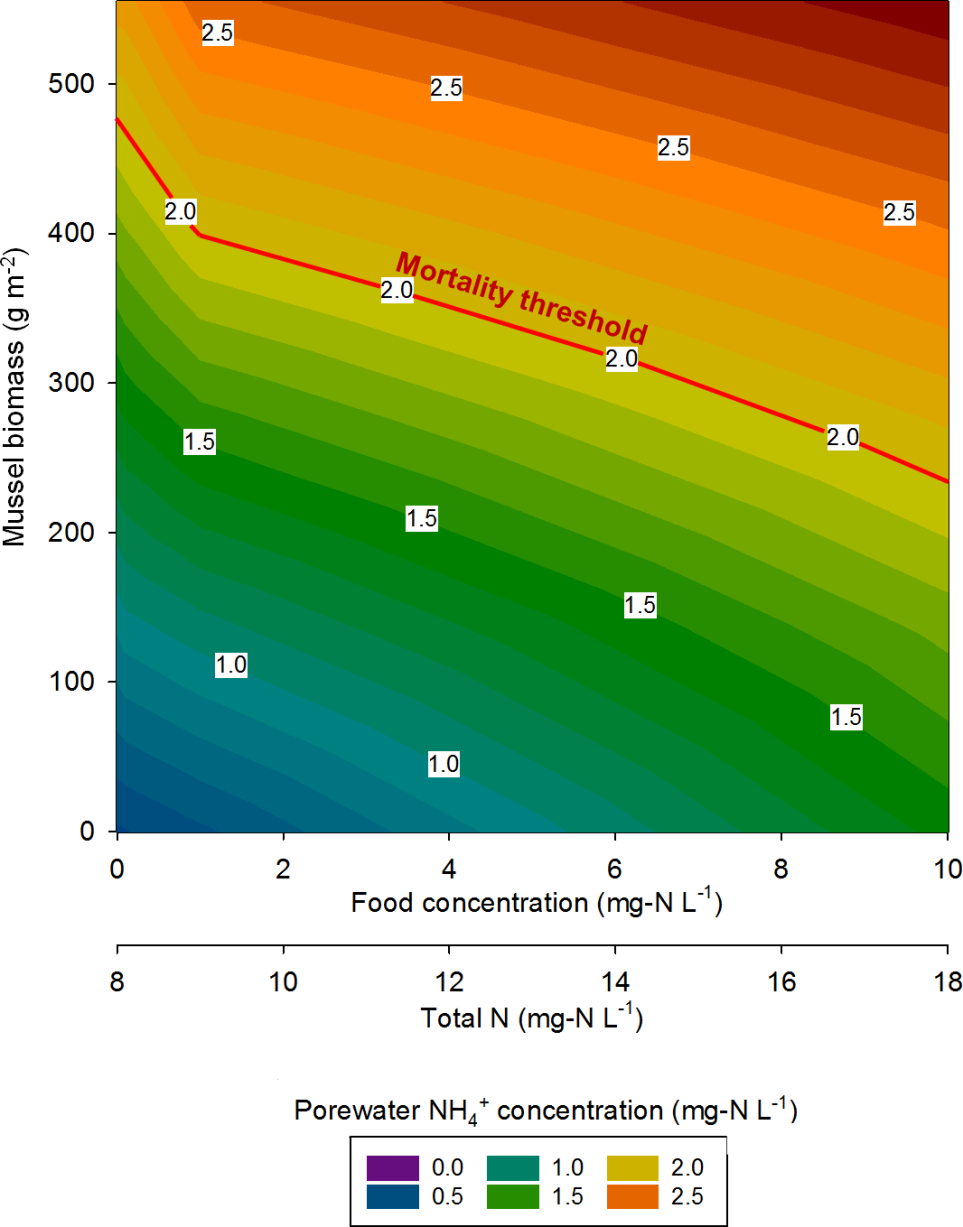
The mussel mortality threshold, defined as a porewater NH${}_{4}^{+}$ concentration of ≥2 mg-N L^−1^ as a function of mussel biomass, overlying water food concentration, and overlying water total N concentration.

At the highest simulated mussel biomass (555 g m^−2^) and the lowest simulated influent water food concentration (0.1 mg-N L^−1^), the porewater NH${}_{4}^{+}$ concentration after a 2,160 h timespan in the absence of mussels, was 0.5 mg-N L^−1^ compared to 2.3 mg-N L^−1^ in the presence of mussels (Fig. 5). The food concentration in mesocosms without mussels was visibly higher than in mescocosms with mussels while NH${}_{4}^{+}$ and NO${}_{2}^{-}$ concentrations in overlying water were lower in the absence of mussels. Mortality threshold contours were estimated by varying mussel biomass and N concentration in the model (Fig. 6). Even when the simulated overlying water food availability was low, the mortality threshold was reached at a mussel biomass of about 480 g m^−2^. At a food concentration of 10 mg-N L^−1^ the mortality threshold was reached at a biomass of about 250 g m^−2^.

In eastern Iowa, the median total N concentration in rivers and streams is commonly >10 mg-N L^−1^ (Kalkhoff et al., 2000), which can place juvenile freshwater mussels at particular risk to ammonia toxicity. Minnesota has a draft criterion for aquatic life of 4.9 mg-N L^−1^ total N, which was exceeded in 68% of samples collected in a study of Iowa waters between 2004 and 2008 (Garrett, 2012). The US EPA national recommended final acute ambient water quality criterion (AWQC) for protecting freshwater organisms from potential effects of ammonia is 17 mg-N L^−1^ and the final chronic AWQC for ammonia is 1.9 mg-N L^−1^ at pH 7.0 and 20 °C (US Environmental Protection Agency, 2013). At a total N concentration of 10 mg L^−1^, our model predicts the mortality threshold to be reached when mussel biomass is about 400 g m^−2^. However, the maximum total N concentration measured between 2004 and 2008 was 37.8 mg-N L^−1^ (Garrett, 2012). Our model suggests the mortality threshold for juvenile *Lampsilis* could be exceeded at low mussel biomass if even a short exposure occurs at such a high total N concentration.

Reflecting on the relationships between nutrients and freshwater mussels conceptualized by Strayer (2014), we concur that high nutrient loads (particularly N in the agricultural Midwest) are a threat to the well-being of mussels. Conversely, our model predicts a somewhat linear mortality threshold relationship as mussel biomass and total N are varied, whereas Strayer stated this relationship would probably be non-linear and non-monotonic. In agreement with Strayer, our model suggests that mussels spatially focus nutrients from the overlying water to the sediments as evidenced by elevated porewater NH${}_{4}^{+}$ in mescosms with mussels. However, our previous work (Bril et al., 2014), and the model developed here, show elevated concentrations of NO${}_{2}^{-}$ and NO${}_{3}^{-}$ in overlying waters as an indirect consequence of mussel activity. This still represents a spatial focusing of nutrients by mussels, but the impact is not seen in the sediment alone.

## Conclusions

The concept of a variable “mussel mortality threshold” as a function of mussel biomass and nutrient loading was successfully explored using a numerical model calibrated with data from mesocosms with and without mussels. With a threshold porewater NH${}_{4}^{+}$ value of 2 mg-N L^−1^, mussel mortality was predicted to occur well within the range of documented total N concentrations in eastern Iowa rivers and streams and at biologically relevant mussel biomasses. The model could be used as a screening tool to determine when mussel populations might be at risk due to high levels of chronic and acute nutrient loadings.

##  Supplemental Information

10.7717/peerj.2838/supp-1 Supplemental Information 1 Supplemental information

## Additional Information and Declarations

### Data Availability

The following information was supplied regarding data availability: The raw data used to run one model simulation is in Figs. 3 and 4. The model equations are in the Supplemental Information.
