# Supplementary material for: Simulated mussel mortality thresholds as a function of mussel biomass and nutrient loading
Source: PeerJ. 2017 Jan 4;5:e2838. doi: 10.7717/peerj.2838 (PMC5217613; doi:10.7717/peerj.2838)
Supplement: Supplemental Information 1 [file peerj-05-2838-s001.docx]

**Supplemental Information**

The differential equations that comprise the numerical model were solved using Euler’s method with 30-min time steps (STELLA version 8.0, ISEE Systems, Inc., Lebanon, New Hampshire).

*Food*. Mussel “food” was modeled to include inputs of photosynthesis and inflow, and outputs of death, settling, respiration/excretion, mussel clearance, and outflow (Equations 1 through 7):

$a_{o,t}=a_{o,t-1}+\frac{a_{r,t}}{\tau}+g-\frac{a_{o,t}}{\tau}-cl-(a_{o,t}\cdot V_{s,a})-ra$ (1)

$a_{p,t}=a_{p,t-1}+(a_{o,t}\cdot V_{s,a})-d$ (2)

$g=k_{g}\cdot a_{o,t}$ (3)

$k_{g}\left( T,N,I \right)=k_{g,T}\cdot\varphi\cdot\varphi_{n,p}$ (3a)

$cl=M_{b}\cdot M_{cl}\cdot a_{o,t}$ (4)

$M_{cl}=k_{cl}\cdot{1.08}^{T_{p}-20}$ (4a)

$V_{s,a}=\frac{0.001}{H}$ (5)

$ra=k_{ra}\cdot a_{o,t}$ (6)

$k_{ra}=0.0004\cdot{1.08}^{T_{o}-20}$ (6a)

$d=k_{d}\cdot a_{p,t}$ (7)

Where, *a_t_* = food concentration at time *t* (mg-N L^-1^) in the overlying (*a*_o_*_,t_*) or porewater (*a_p,t_*), *a_t-1_* = food concentration at time *t-1* (mg-N L^-1^), *a_r.t_* = food concentration in the river water, *τ* = hydraulic retention time (h), *k_g_(T, N, I)* = food photosynthesis rate as a function of temperature, nutrients, and light (h^-1^). The clearance rate of mussels, *M_cl_* (h^-1^ g^-1^ mussel biomass), was defined as the volume of water from which a mussel has filtered all food particles in a given time (Spooner and Vaughn, 2008). This volume was divided by the total mesocosm volume to estimate the fraction of food removed by the total mussel biomass, *M_b_* (grams), over time.

Food photosynthesis, *k_g_(T, N, I)*, was modeled as a function of temperature, nutrients, and light (Equation 3a) where, *k_g,T_* = food growth rate at temperature T (h^-1^), *φ_n_* = nitrogen attenuation factor (dimensionless), *φ_p_* = phosphorus attenuation factor (dimensionless), and *φ_L_* = light attenuation factor (dimensionless). The nitrogen and phosphorus attenuation factors were calculated using the Michaelis-Menten equation with the assumption that the nutrient in shortest supply would control growth. The light attenuation factor was set to 3 times the river phytoplankton biomass to the better capture the phytoplankton sensor data maxima in the mussel mesocosms. In systems less prone to abrupt shifts in food biomass (either actual or as a sensor measurement artifact), actual PAR readings would be converted to the equivalent light attenuation factor (as in Steele, 1965) as a model input.

Additional parameters were *V_s,a_* = food settling rate (m h^-1^), *H* = water depth (m), *k_d_(T)* = temperature-dependent food death rate (h^-1^), *T* = temperature (°C), and *k_ra_(T)* = temperature-dependent food respiration/excretion rate (h^-1^).

*Ammonium-nitrogen.* NH_4_^+^ was modeled to include inputs of org N hydrolysis, food respiration/excretion, mussel excretion, and inflow. Losses were modeled as food uptake, nitrification, and outflow (Equations 8 through 17):

$\frac{\mathrm{dn}_{a,t}}{\mathrm{dt}}=k_{hn}\left( T \right)n_{o,t}+k_{\mathrm{ra}}\left( T \right)a_{t}+M_{\mathrm{ex}}M_{b}+\frac{n_{a,t-1}}{\tau}-F_{\mathrm{am}}k_{g}\left( T,N,I \right)a_{t}-k_{n}\left( T \right)n_{a,t}-\frac{n_{a,t}}{\tau}$ $n_{a,p,t}= n_{a,p,t-1}+hn+D_{n_{a,p,t}}+ex-D_{n_{a,o,t}}$ (8)

$n_{a,o,t}=n_{a,o,t-1}+\frac{n_{n,r,t}}{\tau}+ra+D_{n_{a,o,t}}-\frac{n_{a,o,t}}{\tau}-P_{a}-n-D_{n_{a,p,t}}$ (9)

$hn=k_{hn}\cdot n_{o,p,t}$ (10)

$k_{hn}=0.00004\cdot{1.08}^{T_{p}-20}$ (10a)

$D_{n_{a,p,t}}=k_{D_{n_{a,p,t}}}\cdot n_{a,o,t}$ (11)

$ex=M_{b}\cdot M_{ex}$ (12)

$M_{ex}=k_{e_{M}}\cdot{1.08}^{T_{o}-20}$ (12a)

$D_{n_{a,o,t}}=k_{D_{n_{a,o,t}}}\cdot n_{a,p,t}$ (13)

$ra=k_{ra}\cdot a_{o,t}$ (14)

$k_{ra}=0.0004\cdot{1.08}^{T_{o}-20}$ (14a)

$P_{a}=U_{in}\cdot g$ (15)

$U_{in}=\frac{n_{a,o,t}\cdot n_{n,o,t}}{\left( k_{am}+n_{a,o,t} \right)\cdot\left( k_{am}+n_{n,o,t} \right)}+\frac{k_{am}\cdot n_{a,o,t}}{\left( n_{a,o,t}+n_{n,o,t} \right)\cdot\left( k_{am}+n_{n,o,t} \right)}$ (15a)

$g=k_{g}\cdot a_{o,t}$ (15b)

$k_{g}=k_{g,T}\cdot\varphi\cdot\varphi_{n,p}$ (16)

$n=k_{n}\cdot n_{a,o,t}$ (17)

$k_{n}=0.1\cdot{1.08}^{T_{o}-20}$ (17a)

Where, *n_a,t_* = NH_4_^+^ concentration at time *t* (mg-N L^-1^) in the overlying (*n_a,o,t_*) or porewater (*n_a,p,t_*), *n_a,t-1_* = NH_4_^+^ concentration at time *t-1* (mg-N L^-1^), *hn* = NH_4_^+^ formation in porewater, *k_hn_* = temperature-dependent org N hydrolysis rate (h^-1^), *D_n a,p,t_ =* NH_4_^+^ diffusion from overlying water to porewater, *ex* = NH_4_^+^ excretion by mussels, *M_ex_* = mussel ammonium excretion rate (mg-N L^-1^ h^-1^ g^-1^ mussel biomass), *D_n a,o,t_* = NH_4_^+^ diffusion from porewater to overlying water, *ra* = NH_4_^+^ formation from hydrolysis of food in overlying water, *P_a_* = NH_4_^+^ uptake by food, *U_in_* = food preference for NH_4_^+^ as a nitrogen source (dimensionless), *g =* food growth, *k_g_* = food growth rate, *n* = nitrification of NH_4_^+^ to NO_3_^-^ in the overlying water, and *k_n_(T)* = temperature-dependent nitrification rate (h^-1^).

*Nitrate-nitrogen.* NO_3_^-^ was modeled to include inputs of nitrification and inflow and outputs of plant uptake, denitrification, and outflow (Equations 18 through 29):

$\frac{\mathrm{dn}_{n,t}}{\mathrm{dt}}=k_{n}\left( T \right)n_{a,t}+\frac{n_{n,t-1}}{\tau}-\left( 1-F_{\mathrm{am}} \right)k_{g}\left( T,N,I \right)a_{t}-k_{\mathrm{dn}}\left( T \right)n_{n,t}-\frac{n_{n,t}}{\tau}$ $n_{n,p,t}=n_{n,p,t-1}+D_{n_{n,p,t}}-D_{n_{n,o,t}}-dn$ (18)

$n_{n,o,t}=n_{n,o,t-1}+\frac{n_{n,o,t}}{\tau}+n+D_{n_{n,o,t}}-\frac{n_{n,o,t}}{\tau}-P_{n}-D_{n_{n,p,t}}$ (19)

$D_{n_{n,o,t}}=k_{D_{n_{n,t}}}\cdot n_{n,o,t}$ (20)

$D_{n_{n,p,t}}=k_{D_{n_{n,t}}}\cdot n_{n,p,t}$ (21)

$dn=k_{dn}\cdot n_{n,p,t}$ (22)

$k_{dn}=0.008\cdot{1.08}^{T_{p}-20}$ (23)

$n=k_{n}\cdot n_{a,o,t}$ (24)

$k_{n}=0.1\cdot{1.08}^{T_{o}-20}$ (25)

$P_{n}=\left( 1-U_{in} \right)\cdot g$ (26)

$U_{in}=\frac{n_{a,o,t}\cdot n_{n,o,t}}{(k_{am}+n_{a,o,t})\cdot(k_{am}+n_{n,o,t})}+\frac{k_{am}\cdot n_{a,o,t}}{\left( n_{a,o,t}+n_{n,o,t} \right)\cdot\left( k_{am}+n_{n,o,t} \right)}$ (27)

$g=k_{g}\cdot a_{o,t}$ (28)

$k_{g}=k_{g,T}\cdot\varphi\cdot\varphi_{n,p}$ (29)

Where, *n_n,t_* = NO_3_^-^ concentration at time *t* (mg-N L^-1^) in the overlying (*n_n,o,t_*) or porewater (*n_n,p,t_*), *n_n,t-1_* = NO_3_^-^ concentration at time *t-1* (mg-N L^-1^), *D_n n,p,t_ =* NO_3_^-^ diffusion from overlying water to porewater, *D_n n,o,t_* = NO_3_^-^ diffusion from porewater to overlying water, *dn* = denitrification of NO_3_^-^, *k_dn_* = denitrification rate (h^-1^), *n* = nitrification of NH_4_^+^, *k_n_* = nitrification rate (h^-1^), *P_n_ =* uptake of NO_3_^-^ by food, *U_in_* = food preference for NH_4_^+^ as a nitrogen source (dimensionless), *g =* food growth, and *k_g_* = food growth rate.

*Organic nitrogen.* Org N was modeled to include inputs of phytoplankton death and inflow and outputs of hydrolysis, settling, and outflow (Equations 30 through 35):

$\frac{\mathrm{dn}_{o,t}}{\mathrm{dt}}=k_{d}\left( T \right)a_{t}+\frac{n_{o,t-1}}{\tau}-k_{hn}\left( T \right)n_{o,t}-\frac{V_{s,o}}{H}n_{o,t}-\frac{n_{o,t}}{\tau}$ $n_{o,p,t}=n_{o,p,t-1}+(n_{o,o,t}\cdot V_{s,o})+ d-hn$ (30)

$n_{o,o,t}=n_{o,o,t-1}+\frac{n_{o,r,t}}{\tau}-\frac{n_{o,o,t}}{\tau}-(n_{o,o,t}\cdot V_{s,o})$ (31)

$V_{s,o}=\frac{0.001}{H}$ (32)

$d=k_{d}\cdot a_{p,t}$ (33)

$hn=k_{hn}\cdot n_{o,p,t}$ (34)

$k_{hn}=0.00004\cdot{1.08}^{T_{p}-20}$ (35)

Where, *n_o,t_* = org N concentration at time *t* (mg-N L^-1^) in the overlying (*n_o,o,t_*) or porewater (*n_o,p,t_*), *n_o,t-1_* = org N concentration at time *t-1* (mg-N L^-1^), *V_s,o_* = org N settling rate (m h^-1^), *d* = diffusion of org N out of porewater, *hn* = hydrolysis of org N, and *k_hn_* = org N hydrolysis rate (h^-1^).

*Nitrite-nitrogen.* NO_2_^-^ was modeled to include inputs of nitrification (NH_4_^+^ to NO_2_^-^), denitrification (NO_3_^-^ to NO_2_^-^), and inflow, and outputs of nitrification (NO_2_^-^ to NO_3_^-^), denitrification (NO_2_^-^ to N_2_ gas), and outflow (Equations 36 through 47):

$\frac{\mathrm{dn}_{i,t}}{\mathrm{dt}}=k_{\mathrm{ai}}\left( T \right)n_{a,t}+k_{\mathrm{ni}}\left( T \right)n_{n,t}+\frac{n_{i,t-1}}{\tau}-k_{\mathrm{in}}\left( T \right)n_{i,t}-k_{\mathrm{ig}}\left( T \right)n_{i,t}-\frac{n_{i,t}}{\tau}$ $n_{i,p,t}=n_{i,p,t-1}+ni+D_{n_{i,p,t}}-ig-D_{n_{i,o,t}}$ (36)

$n_{i,o,t}=n_{i,o,t-1}+\frac{n_{i,r,t}}{\tau}+ai+D_{n_{i,o,t}}-\frac{n_{i,o,t}}{\tau}-in-D_{n_{i,p,t}}$ (37)

$ni=k_{ni}\cdot n_{n,p,t}$ (38)

$k_{ni}=0.008\cdot{1.08}^{T_{p}-20}$ (39)

$D_{n_{i,p,t}}=k_{D_{n_{i,t}}}\cdot n_{i,o,t}$ (40)

$D_{n_{i,o,t}}=k_{D_{n_{i,t}}}\cdot n_{i,p,t}$ (41)

$ig=k_{ig}\cdot n_{i,p,t}$ (42)

$k_{ig}=0.07\cdot{1.08}^{T_{p}-20}$ (43)

$ai=k_{ai}\cdot n_{a,o,t}$ (44)

$k_{ai}=0.12\cdot{1.08}^{T_{o}-20}$ (45)

$in=k_{in}\cdot n_{i,o,t}$ (46)

$k_{in}=0.21\cdot{1.08}^{T_{p}-20}$ (47)

Where, *n_i,t_* = NO_2_^-^ concentration at time *t* (mg-N L^-1^) in the overlying (*n_i,o,t_*) or porewater (*n_i,p,t_*), *n_i,t-1_* = temperature-dependent NO_2_^-^ concentration at time *t-1* (mg-N L^-1^), *ni* = NO_3_^-^ to NO_2_^-^ denitrification, *k_ni_(T)* = temperature-dependent NO_3_^-^ to NO_2_^-^ denitrification rate (h^-1^), *D_n i,p,t_* = diffusion of overlying water NO_2_^-^ to porewater, *D_n i,o,t_* = diffusion of porewater NO_2_^-^ to the overlying water, *ig* = NO_2_^-^ to N_2_ gas denitrification, *k_ig_(T)* = NO_2_^-^ to N_2_ gas denitrification rate (h^-1^), *ai* = NH_4_^+^ to NO_2_^-^ nitrification, *k_ai_(T)* = temperature-dependent NH_4_^+^ to NO_2_^-^ nitrification rate (h^-1^), *in* = NO_2_^-^ to NO_3_^-^ nitrification, and *k_in_(T)* = temperature-dependent NO_2_^-^ to NO_3_^-^ nitrification rate (h^-1^).

*Total nitrogen.* Total N was modeled by adding together the model results for NH_4_^+^, NO_3_^-^, org N, and NO_2_^-^ at each time step (Equation 48):

 (48)

Where, *n_total,t_* = total N concentration at time *t* (mg-N L^-1^).
